# [^18^F]Mefway: Imaging Serotonin 5HT_1A_ Receptors in Human Postmortem Alzheimer’s and Parkinson’s Disease Anterior Cingulate. Potential Applications to Human Positron Emission Tomography Studies

**DOI:** 10.3390/biom15040592

**Published:** 2025-04-16

**Authors:** Noresa L. Gonzaga, Fariha Karim, Christopher Liang, Jogeshwar Mukherjee

**Affiliations:** Preclinical Imaging, Department of Radiological Sciences, University of California-Irvine, Irvine, CA 92697, USA; nlgonzag@uci.edu (N.L.G.); fkarim1@uci.edu (F.K.); liangc@uci.edu (C.L.)

**Keywords:** [^18^F]mefway, [^18^F]fallypride, serotonin receptors, dopamine receptors, Parkinson’s disease, Alzheimer’s disease

## Abstract

Serotonin 5HT_1A_ receptors may be affected in neurodegeneration, such as Alzheimer’s disease (AD) and Parkinson’s disease (PD). Using the selective 5HT_1A_ receptor positron emission tomography (PET) imaging agent, [^18^F]mefway, autoradiographic studies from postmortem human brains of AD, PD, and cognitively normal (CN) subjects were carried out. Levels of [^18^F]mefway binding were compared with monoamine oxidase A (MAO-A) measured using [^18^F]FAZIN3 binding and dopamine D2/D3 receptors measured using [^18^F]fallypride binding in the same subjects. Autoradiograms of brain sections of the anterior cingulate and corpus callosum from CN, PD, and AD subjects (*n* = 6 in each group) were analyzed. Significant increased binding of [^18^F]mefway was found in the AD (+30%) and PD (+11%) brains compared to CN brains. This increase positively correlated to increased [^18^F]FAZIN3 binding, suggesting greater 5HT_1A_ receptor availability when MAO-A levels are higher. Differences in [^18^F]fallypride binding in the three groups were not significant. Our results support the finding that the availability of 5HT_1A_ receptors in AD and PD is elevated in the anterior cingulate cortex and is negatively correlated with MAO-A. This upregulation may potentially be a response to lower serotonin levels due to the increased levels of MAO-A activity in this brain region or other neuroinflammatory changes. Thus, 5HT_1A_ receptors may be a potential target for diagnostic and therapeutic approaches for AD and PD.

## 1. Introduction

Serotonin and associated serotonin 5HT_1A_ receptors have attracted attention due to their potential role in neurodegeneration and therapeutics development [1,2,3]. Therapeutic efforts for Alzheimer’s disease (AD) [4,5] and Parkinson’s disease (PD) [6,7,8,9] targeting the serotonin 5HT_1A_ receptors have been underway. Postmortem human AD brain studies suggest no change in 5HT_1A_ receptor availability in the temporal cortex of AD subjects without aggressive behavior [10], while a reduction of 5HT_1A_ receptor availability was seen in the AD dentate gyrus [11]. Noninvasive imaging agents for use in positron emission tomography (PET) studies of serotonin 5HT_1A_ receptor availability have been pursued in order to help understand various brain disorders [12]. Previous PET studies on AD and mild cognitive impairment (MCI) using the 5HT_1A_ agent, [^18^F]MPPF, suggest a decrease of 5HT_1A_ receptor availability in the hippocampus of mild cognitive impairment (MCI) patients and a greater decrease in AD patients [13]. However, other PET studies using [^18^F]MPPF showed little change or an increase of 5HT_1A_ receptor availability in the inferior temporal and occipital gyrus in amnestic MCI [14,15]. Using [^11^C]WAY-100635, a reduction of 5HT_1A_ receptor availability was found in the dorsal raphe of Parkinson’s disease patients [16]. Although imaging findings of serotonin 5HT_1A_ receptor availability have been mixed, the relevance to learning, memory, and neuroplasticity clearly emphasizes the need to further evaluate their role in both AD and PD [17,18,19,20].

**Figure 1:**
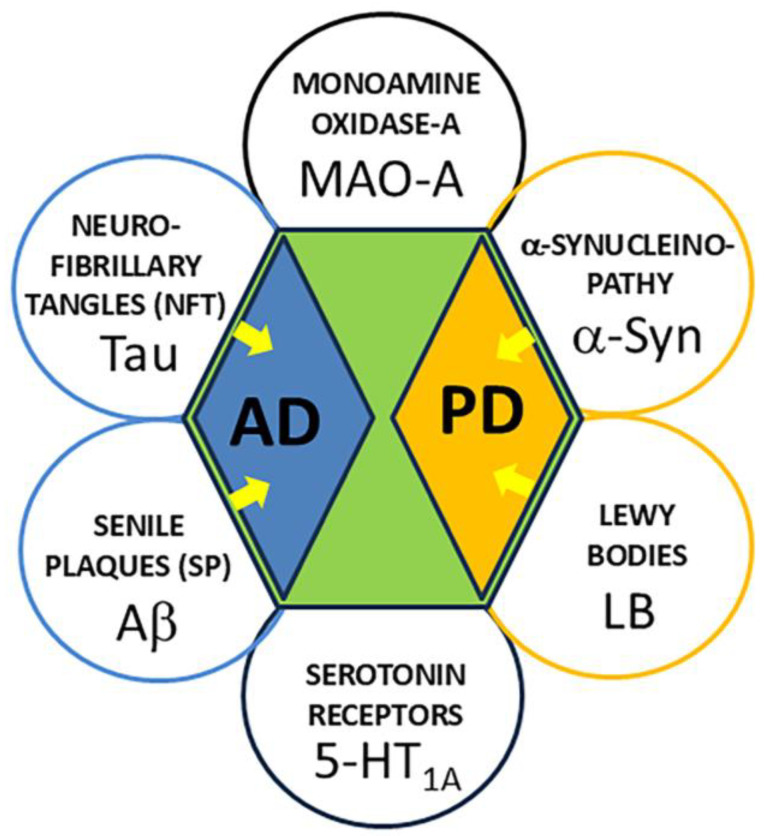
Schematic showing biomarkers for AD and PD. The yellow arrows indicate the known relationships between biomarkers and their respective neurodegenerative disease. Tau (neurofibrillary tangles, NFT) PET imaging agents are currently used for in AD subjects. Aβ-plaque (senile plaques, SP) PET imaging agents are currently used in AD subjects. α-Synuclein aggregates are a potential biomarker in PD. Lewy bodies are a potential biomarker for PD. Monoamine oxidase-A (MAO-A) in AD and PD has been identified as a potential new biomarker. Serotonin 5HT_1A_ receptors in AD and PD is investigated in this report.

PET imaging of the two biomarkers (amyloid β, Aβ plaques, and neurofibrillary tangles, NFT) is now playing a major role in the staging of AD and in the development of treatment evaluations of AD [21,22,23,24] (Figure 1). Neuroinflammation in AD is now regarded as an early indicator of disease. We have recently reported increases in monoamine oxidase-A (MAO-A) levels measured autoradiographically using [^18^F]FAZIN3 in postmortem AD brain slices of anterior cingulate [25]. This suggested a relationship between the accumulation of Aβ plaques and Tau, with increases in [^18^F]FAZIN3 binding. In addition to MAO-A, the aggregate effect of proteinopathies and upregulation of MAO-A on serotonergic neurotransmission in the anterior cingulate regions in the human brain needs to be evaluated.

Disruption of cellular functions, α-synuclein aggregation, Lewy bodies inducing mitochondrial damage and deficits, and synaptic dysfunctions all contribute to an inflammatory component in PD (Figure 1). We have recently reported increases in MAO-A levels measured autoradiographically using [^18^F]FAZIN3 in postmortem PD brain slices of anterior cingulate [26]. Since MAO-A is responsible for the degradation of monoaminergic neurotransmitters such as serotonin and others, components of this neurotransmitter-receptor system may be altered in PD.

**Figure 2:**
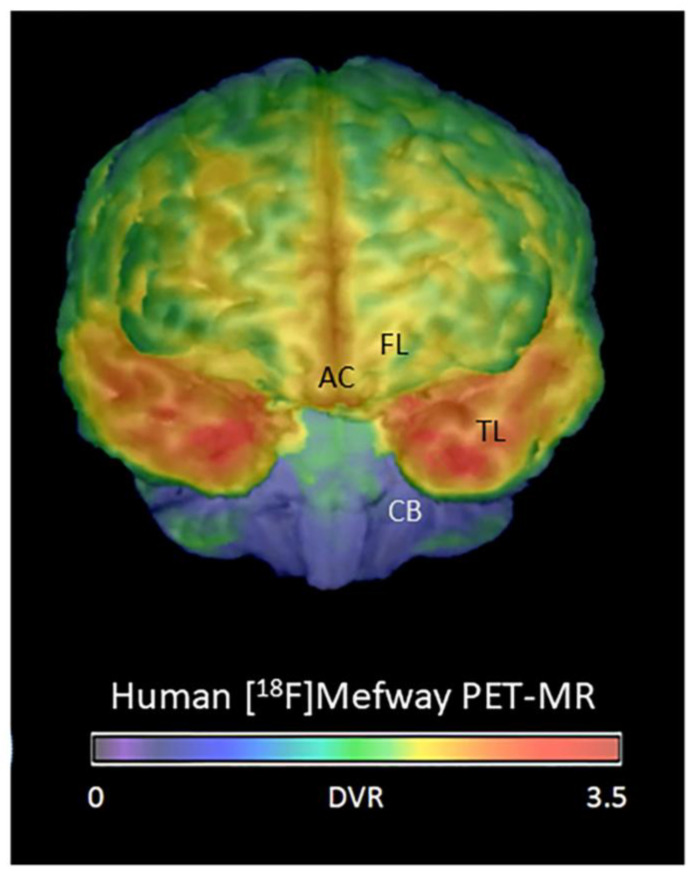
[^18^F]mefway for imaging human serotonin 5HT_1A_ receptors. Brain surface image of [^18^F]mefway PET-MRI from cognitively normal subject showing binding of [^18^F]mefway. High binding of [^18^F]mefway is seen in the temporal lobe (TL; red) which includes the hippocampus, moderate binding in frontal lobe (FL; yellow) and anterior cingulate (AC; yellow red) and least in cerebellum (CB; blue).

Amongst the several PET imaging agents reported for quantitative imaging of 5HT_1A_ receptors in CNS disorders [12], [^18^F]mefway (Figure 2) has been successfully used in human studies [27,28,29]. Brain regions rich in 5HT_1A_ receptors include the hippocampus and temporal cortex, while other cortical regions, including the anterior cingulate cortex, also have significant amounts of 5HT_1A_ receptors, as revealed by [^18^F]mefway binding (Figure 2).

Using autoradiographic imaging methods, the goal of this work was to evaluate binding of [^18^F]mefway in postmortem human PD, AD, and cognitively normal (CN) brain sections of the anterior cingulate for measuring changes in 5HT_1A_ receptors. Since [^3^H]]WAY-100635 has been used in previous autoradiographic studies of 5HT_1A_ receptors [30], we carried out initial experiments in CN and AD frontal cortex. Because of the advantages of fluorine-18 over tritium, [^18^F]mefway was chosen for comparative studies of CN, AD, and PD. We also evaluated dopamine D2/D3 receptors in the same subjects using the high-affinity PET imaging agent [^18^F]fallypride [31]. Levels of the two receptor subtypes were compared with our previously reported measures of MAO-A using [^18^]FAZIN3. The human anterior cingulate plays an important role in cognitive function [32,33] and was chosen for the present study. It shows abundant accumulation of Aβ plaques [34] and tau [35] in AD patients. Microglial activity in the anterior cingulate in major depressive disorder has been reported [36]. The presence of these biomarkers in the anterior cingulate and its role in cognitive function [37] led to this brain region being chosen for the present study.

## 2. Materials and Methods

### 2.1. General Methods

Iodine-125 sodium iodide was purchased from American Radiolabeled Chemicals, Inc., St. Louis, MO, USA, and fluorine-18 was purchased from PETNET, Inc. (Knoxville, TN, USA). The specialty chemical raclopride was purchased (Sigma Aldrich, St. Louis, MO, USA). [^18^F]mefway [38] and [^18^F]fallypride [39] were produced in house. [^3^H]WAY-100635 (methoxy ^3^H, 2.74TBq/mmol; Amersham, UK) was used for autoradiographic studies.

### 2.2. Postmortem Human Brain

Human postmortem brain tissue samples were obtained from the Banner Sun Health Research Institute (BHRI), Sun City, AZ, USA, brain tissue repository for in vitro experiments. Well-characterized frozen brain samples were obtained from BHRI, Sun City Arizona (Table 1). Brain tissue samples from CN, AD, and PD subjects were selected by observing the presence and absence of end-stage pathology. Horizontal slices were cut 10-μm thick using a Leica 1850, cryotome (Deer Park, IL, USA) at -20^o^C. The brain slices contained the frontal cortex, anterior cingulate, and corpus callosum regions (CN, *n* = 6; ages 81–90 and AD, *n* = 6, ages 64–89; Table 1). Brain sections were stored at −80 °C. All postmortem human brain studies were approved by the Institutional Biosafety Committee of University of California, Irvine (protocol # BUA-R144, date: 4 January 2024).

### 2.3. [^3^H]WAY 100635 for Serotonin 5HT-_1A_R Imaging

All brain slices were preincubated in 50 mM Tris-HCl buffer (pH 7.6) for 10 min. Brain slices from AD and CN frontal cortex were incubated with [^3^H]WAY-100635 (1 nM) was incubated in the assay buffer (50 mM Tris-HCl, pH 7.6). Nonspecific binding was determined by including 10 μM of WAY-100635. The brain slices were incubated for 1 h in a 22 °C water bath. After incubation, slides were washed twice with Tris buffer (each wash lasting 5 min) and once with ice-cold water (2 min). Brain sections were air-dried, exposed (7 days) on tritium phosphor screens, and then scanned on the Phosphor Autoradiographic Imaging System (Packard Instruments Co., Boston, MA, USA). Using the Optiquant acquisition and analysis program (Packard Instruments Co., Boston, MA, USA), regions of interest were drawn in the gray matter and white matter regions. Digital light units/mm^2^ (DLU/mm^2^) were used to quantify the extent of binding.

### 2.4. [^18^F]Mefway for Serotonin 5HT-_1A_R Imaging

Brain slices were preincubated in 50 mM Tris-HCl buffer (pH 7.6) for 10 min. The slices were then incubated with 74 kBq/mL of [^18^F]mefway at 22 °C for 1 h. Nonspecific binding was measured in the presence of 10 μM of WAY-100635. After incubation, slides were washed as described above in Section 2.3. Slides were then air-dried, and exposed to a phosphor screen for 24 h. Analysis was carried out as described above in Section 2.7.

### 2.5. [^18^F]Fallypride for Dopamine D2/D3 Receptor Imaging

Binding studies using [^18^F]fallypride were carried out on adjacent brain slices of anterior cingulate. Using tris buffer (50 mM Tris HCl, 2.5 mM CaCl_2_, 125 mM NaCl, 1 mM MgCl, 5 mM KCl, 0.l mM sodium ascorbate, pH 7.4) sections were preincubated in at room temperature for 10 min. Incubation in the buffer at room temperature for 10 min followed by incubation in buffer with 37 kBq/mL [^18^F]fallypride at 22 °C for 1 h was carried out. Nonspecific binding was measured in the presence of 10 μM raclopride. After incubation, slides were washed as described above in Section 2.3. Slides were air dried, and exposed to a phosphor screen for 24 h. Analysis was carried out as described above in Section 2.7.

### 2.6. Immunohistochemistry

Immunostaining of all brain sections was carried out by University of California-Irvine, Pathology services as previously described [26,41,42].

### 2.7. Image Analysis

#### 2.7.1. [^3^H]WAY 100635 

Regions of interest (ROI) in the frontal cortex autoradiographic images of [^3^H]WAY 100635 were quantified using measurements of DLU/mm^2^. Gray matter (GM) and WM binding in the AD and CN subjects were compared. Group differences between AD and CN subjects were evaluated using *t*-tests.

#### 2.7.2. [^18^F]Mefway

Regions of anterior cingulate (GM) and corpus callosum (WM) autoradiographic images of [^18^F]mefway were quantified similarly. Binding in GM and WM binding in the AD, PD and CN subjects were compared. Group differences between AD, PD, and CN subjects were evaluated using *t*-tests.

#### 2.7.3. [^18^F]Fallypride

Anterior cingulate and corpus callosum autoradiographic images of [^18^F]fallypride were quantified using measurements of DLU/mm^2^. Binding in GM and WM in AD, PD and CN subjects were compared and differences were evaluated using *t*-tests.

#### 2.7.4. [^18^F]FAZIN3

Our previously reported ROI analysis in the anterior cingulate and corpus callosum autoradiographic images of [^18^F]FAZIN3 was used [26,27]. Group differences between AD, PD and CN subjects were evaluated using *t*-tests.

#### 2.7.5. [^125^I]IBETA and [^125^I]IPPI

Analysis in the anterior cingulate and corpus callosum autoradiographic images of [^125^I]IBETA and [^125^I]IPPI was used [26,41].

### 2.8. Statistical Analysis

Group differences between AD and CN subjects were assessed using the average GM/WM ratios and were determined using Microsoft Excel 16 and GraphPad Prism 10.4.2. The statistical power was determined with Student’s *t*-test, and *p* values of <0.05 were considered to indicate statistical significance. Spearman’s correlation was carried out in certain cases. The linear correlations and ANOVA analysis of the bindings between the different radiotracers were used to evaluate potential relationships between the different biomarkers.

## 3. Results

### 3.1. Postmortem Human CN, PD and AD Brains

Table 1 describes the characteristics of the CN, PD, and AD subjects used in the study. Brain slices (10 μM thick) of the subjects were immunostained with anti-Aβ, anti-Tau, anti-ubiquitin and anti-α-synuclein to confirm the presence of Aβ plaques and NFT for AD and LB and α-synuclein aggregates for PD [26,27,42]. Adjacent brain slices from the subjects were used for autoradiographic studies with [^18^F]mefway for serotonin 5HT_1A_ receptors and [^18^F]fallypride for dopamine D2/D3 receptors, similar to our previously reported studies on these subjects using the MAO-A radiotracer, [^18^F]FAZIN3 [26,27].

### 3.2. Frontal Cortex Serotonin 5HT1A Receptors

**Figure 3:**
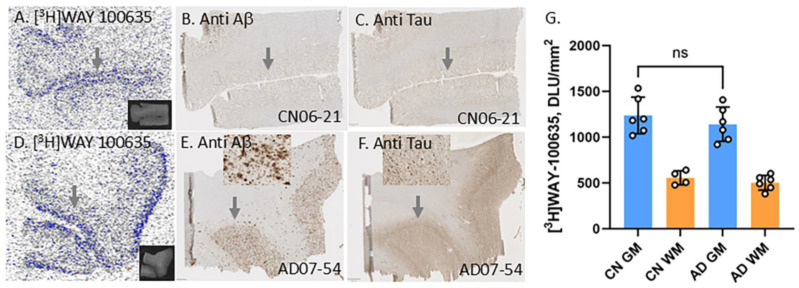
[^3^H]WAY 100635 imaging in AD brain frontal cortex: Arrows indicate the same location on each adjacent brain slice. (**A**) [^3^H]WAY 100635 binding to serotonin 5HT_1A_ receptors in the GM of control subject CN06-21. Nonspecific binding is seen in the WM regions (inset shows corresponding brain slice 10 μm thick of CN06-21). (**B**) Anti-Aβ immunostaining of CN06-21 confirmed absence of Aβ plaques. (**C**) Anti-Tau immunostaining of CN06-21 confirmed absence of NFT. (**D**) [^3^H]WAY 100635 binding to serotonin 5HT_1A_ receptors in the GM of AD subject AD07-54. Nonspecific binding is seen in the WM regions (inset shows corresponding brain slice 10 μm thick of AD07-54). (**E**) Anti-Aβ immunostaining of AD07-54 confirmed presence of Aβ plaques (inset). (**F**) Anti-Tau immunostaining of AD07-54 confirmed presence of NFT (inset). (**G**) Plot shows no significant (ns) difference between the averages of [^3^H]WAY 100635 binding in GM and WM between all CN and AD subjects FC.

Initial binding of [^3^H]WAY-100635 was carried out in the frontal cortex of a limited number of subjects in order to evaluate extent of binding. Previous studies using [^11^C]WAY-100635 and [^3^H]WAY-100635 have been reported to exhibit hippocampal and cortical binding [30]. Figure 3 shows the presence of specific binding in the cortical layers in both AD and CN subjects using [^3^H]WAY-100635. White matter did not exhibit any specific binding of [^3^H]WAY 100635. As expected, the binding in the frontal cortex gray matter was low, which was consistent with our in vivo PET findings using [^18^F]mefway (FL; Figure 2). Distribution across the cortical layers was uneven, with higher binding in the outer cortical layers. This is consistent with previous reports on the distribution of serotonin 5HT1A receptors in the cortex [30]. In this small group of subjects (*n* = 6), there was no significant difference in the levels of [^3^H]WAY 100635 binding in the AD versus CN subjects. Because AC has higher levels of [^18^F]mefway binding (AC; Figure 2), suggesting greater amount of serotonin 5HT1A receptors compared to the frontal cortex, our subsequent studies turned to AC and [^18^F]mefway binding.

### 3.3. [^18^F]Mefway Imaging in AD Anterior Cingulate

**Figure 4:**
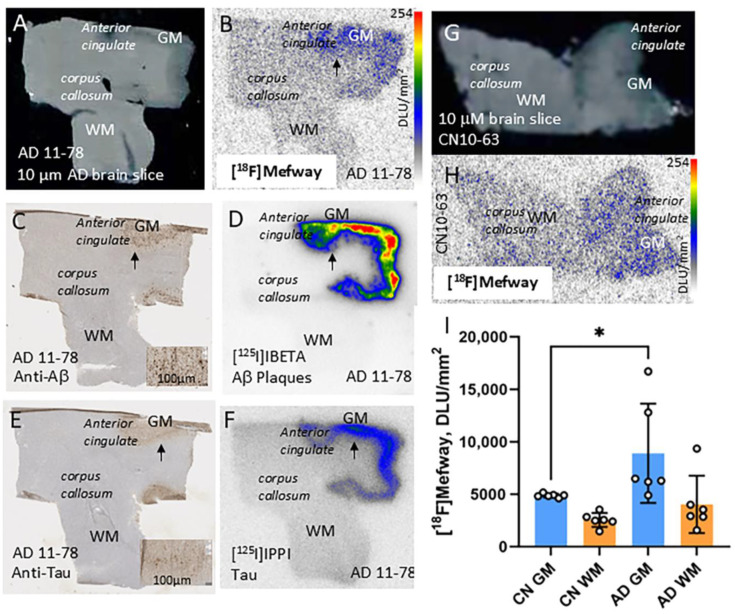
[^18^F]Mefway imaging in AD anterior cingulate: (**A**) Brain slice of AD 11-78 showing GM (AC) and WM (CC) regions. (**B**) Binding of [^18^F]mefway in the adjacent slice of AD 11-78 subject shows preferential binding in the GM (arrow). (**C**) Anti-Aβ immunostaining of AD 11-78 confirmed presence of Aβ plaques (arrow and inset). (**D**) [^125^i]IBETA binding to Aβ plaques was observed in the GM of the adjacent slice of the AD 11-78 subject (arrow). (**E**) Anti-Tau immunostaining of AD 11-78 confirmed presence of NFT (arrow and inset). (**F**) [^125^I]IPPI binding to tau was observed in the GM of the adjacent slice of the AD 11-78 subject (arrow). (**G**) Brain slice of CN 10-63 showing GM (AC) and WM (CC) regions. (**H**) Binding of [^18^F]mefway in GM in the adjacent slice of CN 10-63 subject shows preferential binding. (**I**) Plot shows significant difference (* = *p* < 0.05) between the averages of [^18^F]mefway binding in all CN and AD subjects, with higher levels of binding in AD.

Figure 4 shows binding of [^18^F]mefway in the anterior cingulate brain sections in AD and CN subjects. Binding of [^18^F]mefway in the brain slice of AD 11-78 subject shows preferential binding in the GM (Figure 4B). Anti-Aβ IHC of AD 11-78 subject confirmed the presence of numerous Aβ plaques in the GM regions of the anterior cingulate (Figure 4C), which was further confirmed by [^125^I]IBETA labeling of Aβ plaques (Figure 4D). Additionally, anti-Tau immunostaining of AD 11-78 confirmed presence of NFT (Figure 4E) and [^125^I]IPPI binding to tau was observed in the GM of the adjacent slice of the AD 11-78 subject (Figure 4F). Brain slice of CN 10-63 showed GM (AC) and WM (CC) regions with lower levels of [^18^F]mefway binding. A significant difference (*p* < 0.05) between the averages of [^18^F]mefway binding was present in all CN and AD subjects, with higher levels of binding in AD.

### 3.4. [^18^F]Mefway Imaging in PD Anterior Cingulate

**Figure 5:**
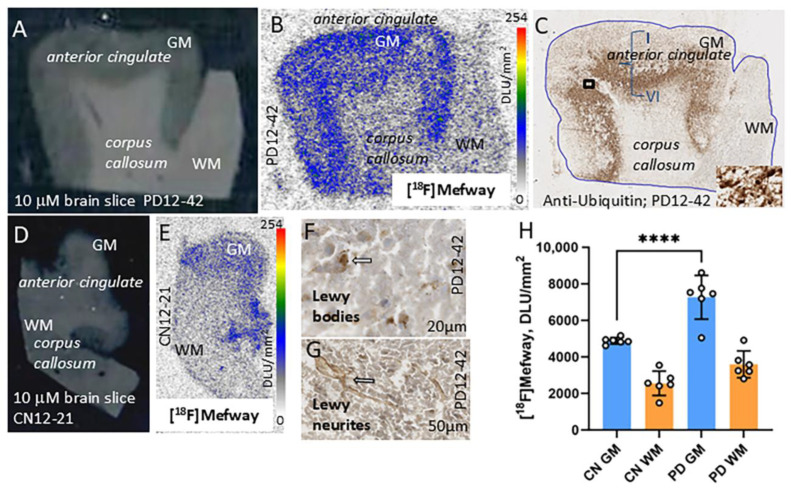
[^18^F]Mefway imaging in PD anterior cingulate: (**A**) Brain slice (10 μm) of PD subject (PD 12-42) showing GM region of anterior cingulate and WM region of corpus callosum. (**B**) [^18^F]Mefway binding to serotonin 5HT_1A_ receptors in the GM of subject PD 12-42. Nonspecific binding is seen in the WM regions. (**C**) Brain slice from PD subject 12-42 stained with anti-ubiquitin (UIHC) showing Lewy body (LB) in the GM regions (inset shows close up of LB in square box). (**D**) Brain slice (10 μm) from CN subject 12-21 showing GM region of anterior cingulate and WM region of corpus callosum. (**E**) [^18^F]Mefway binding to serotonin 5HT_1A_ receptors in the GM of subject CN 12-21. Nonspecific binding is seen in the WM regions. (**F**) Magnified view of GM showing anti-α-synuclein labeled Lewy body (indicated by arrow) in the PD brain slice. (**G**) Magnified view of GM showing anti-α-synuclein labeled Lewy neurites (indicated by arrow) in the PD brain slice. (**H**) Plot shows averages of all PD and CN subjects with higher [^18^F]mefway binding in PD (“**** = *p* < 0.0001” unpaired two-tailed *t*-test).

Distinct [^18^F]mefway binding to serotonin 5HT_1A_ receptors in the GM of subject PD 12-42 was observed with lower levels in WM (Figure 5A,B). The binding of [^18^F]mefway corresponded to anti-ubiquitin (UIHC)-stained Lewy body (LB) in the GM regions (Figure 5C). Although LB were more abundant in cortical layers IV-VI, [^18^F]mefway binding occurred in the outer and lower cortical layers more than in the middle layers. This is similar to that reported for [^3^H]WAY 100635 [30]. Magnified view of GM showing anti-α-synuclein-labeled LB and anti-α-synuclein-labeled Lewy neurites in the PD brain slice of PD 12-42 (Figure 5D,E). Lower levels of [^18^F]mefway binding were observed in the CN subject’s 12-21 GM region of the anterior cingulate and WM region of the corpus callosum (Figure 5F,G). Figure 5H shows averages of all PD and CN subjects with higher [^18^F]mefway binding in PD (“**** = *p* < 0.0001” unpaired two-tailed *t*-test).

The average ratio of GM/WM in the six AD subjects was 2.48. The ratio of GM/WM in the six CN subjects averaged 1.90. When comparing the GM/WM ratios, a 30% increase in [^18^F]mefway binding was observed in AD. The average ratio of GM/WM in the six PD subjects was 2.10, suggesting an 11% increase in PD compared to CN. These findings suggest an increase in available 5HT_1A_ receptors in AD and PD.

### 3.5. [^18^F]Mefway and [^18^F]FAZIN3 Comparison in PD and AD Anterior Cingulate

**Figure 6:**
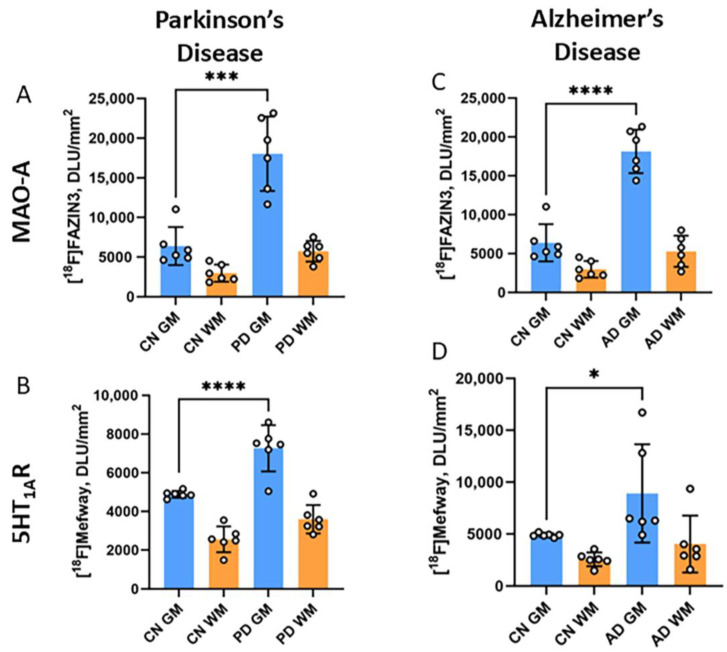
MAO-A ([^18^F]FAZIN3) and 5HT_1A_R ([^18^F]mefway) in PD and AD: (**A**) [^18^F]FAZIN3 binding to MAO-A in all the PD subjects and CN subjects showing higher binding in PD (“*** = *p* < 0.001” unpaired two-tailed *t*-test). (**B**) [^18^F]Mefway binding to serotonin 5HT_1A_ in all the PD subjects and CN subjects showing higher binding in PD (“**** = *p* < 0.0001” unpaired two-tailed *t*-test); (**C**). [^18^F]FAZIN3 binding to MAO-A in all the AD subjects and CN subjects showing higher binding in AD (“**** = *p* < 0.001” unpaired two-tailed *t*-test). (**D**) [^18^F]Mefway binding to serotonin 5HT_1A_ in all the AD subjects and CN subjects showing higher binding in PD (“* = *p* < 0.05” unpaired two-tailed *t*-test).

[^18^F]FAZIN3 binds reversibly and selectively to MAO-A in CN, AD, and PD subjects [26,27]. Our previous studies reported higher levels of significant binding of [^18^F]FAZIN3 in the anterior cingulate of both AD and PD brains (Figure 6A,C). These findings suggest a plausible effect on the degradation of monoamines, such as serotonin, in brain regions. Such neurotransmitter depletion may upregulate neuroreceptors, such as 5HT_1A_ receptors. Our current findings show increased [^18^F]mefway binding in both AD and PD, which corresponded to increases in [^18^F]FAZIN3 (Figure 6). Changes in AD were higher compared to PD. Thus, it appears that increased availability of MAO-A results in increased 5HT_1A_ receptor availability.

### 3.6. [^18^F]Fallypride Imaging in PD and AD Anterior Cingulate

**Figure 7:**
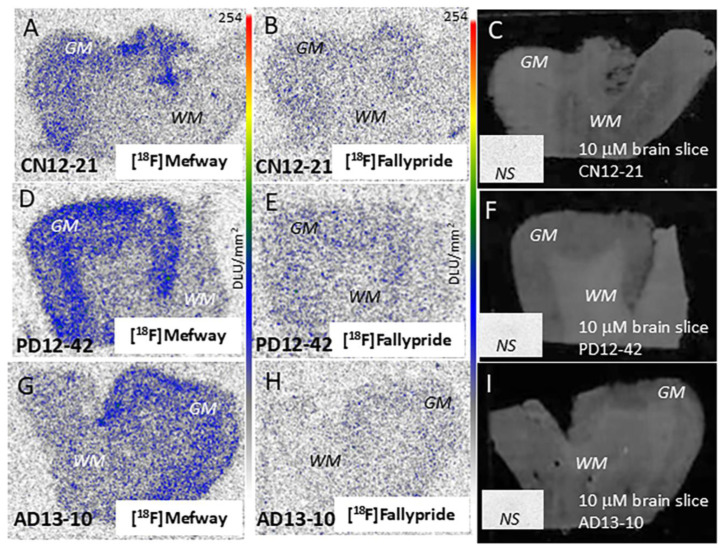
Comparison of [^18^F]mefway and [^18^F]fallypride in CN, PD, and AD subjects: (**A**–**C**) CN subject CN 12-21 showing [^18^F]mefway binding (**A**), [^18^F]fallypride binding (**B**), and scan of the corresponding brain slice. Inset shows nonspecific binding of [^18^F]fallypride in the presence of 10 μM raclopride (**C**); Autoradiography scale bar: 0–254 digital light units (DLU)/mm^2^. (**D**–**F**) PD subject PD 12-42 showing [^18^F]mefway binding (**D**), [^18^F]fallypride binding (**E**), and scan of the corresponding brain slice. Inset shows nonspecific binding of [^18^F]fallypride in the presence of 10 μM raclopride (**F**); (**G**–**I**). AD subject AD 13-10 showing [^18^F]mefway binding (**G**), [^18^F]fallypride binding (**H**) and scan of the corresponding brain slice. Inset shows nonspecific binding of [^18^F]fallypride in the presence of 10 μM raclopride (**I**). Autoradiography scale bar: 0–254 digital light units (DLU)/mm^2^.

Dopamine receptors were assessed using [^18^F]fallypride in the CN, AD, and PD subjects. Levels of [^18^F]fallypride in the human anterior cingulate are low [31]. However, measurable levels of [^18^F]fallypride binding to dopamine receptors were found in the brain slices of CN, AD, and PD (Figure 7B,E,H), which were confirmed by displacement studies with raclopride (inset in Figure 7C,F,I). Figure 7 shows the higher binding of [^18^F]mefway compared to [^18^F]fallypride in the same CN, AD, and PD subjects.

**Figure 8:**
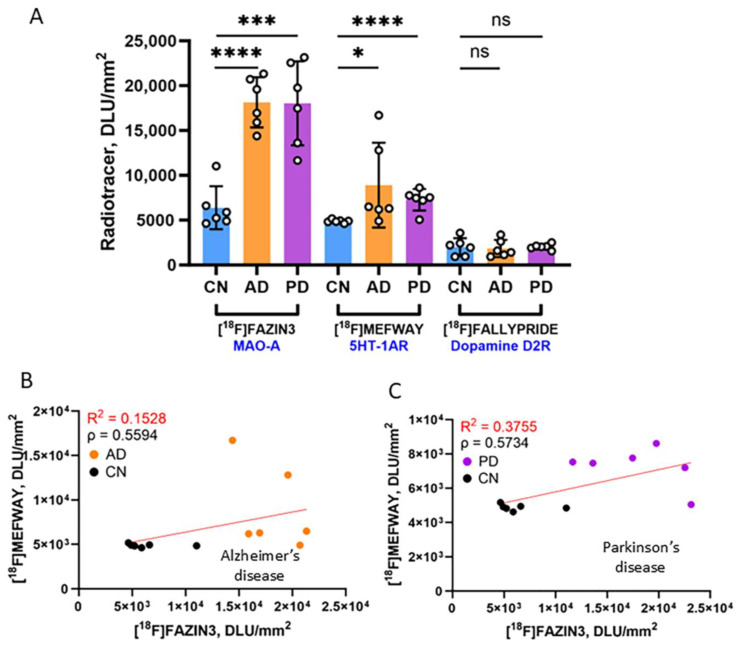
Comparison of biomarkers: (**A**) Plot shows average binding of three radiotracers to anterior cingulate brain region in CN, AD and PD subjects. Binding of [^18^F]FAZIN3 to MAO-A was significantly increased in AD and PD subjects compared to CN subjects. Similarly, binding of [^18^F]mefway to serotonin 5HT_1A_ receptors was also significantly increased in both AD and PD subjects. Binding of [^18^F]fallypride to dopamine D2/D3 receptors was not affected in AD and PD compared to CN subjects. T test *p* values: ns *p* > 0.05; * *p* ≤ 0.05; *** *p* ≤ 0.001. **** = *p* < 0.0001; (**B**) Correlation of [^18^F]FAZIN3 binding with [^18^F]mefway binding in CN (black circles) and AD subjects (orange circles) (R^2^ = 0.1528; Spearman’s correlation ρ = 0.5594); (**C**). Correlation of [^18^F]FAZIN3 binding with [^18^F]mefway binding in CN (black circles) and PD subjects (purple circles) (R^2^ = 0.3755; Spearman’s correlation ρ = 0.5734).

Shown in Figure 8A is the comparison of the binding of [^18^F]FAZIN3, [^18^F]mefway and [^18^F]fallypride in the GM of all the subjects. Highest binding of [^18^F]FAZIN3 was observed in all three groups of subjects. Both AD and PD had significant increases in the binding of [^18^F]FAZIN3, suggesting higher MAO-A levels. Levels of [^18^F]mefway binding were lower when compared to [^18^F]FAZIN3. However, compared to CN, levels of [^18^F]mefway binding were higher in both AD and PD, suggesting a greater availability of serotonin 5HT1A receptors. There was a positive correlation of [^18^F]mefway with [^18^F]FAZIN3 in AD (Figure 8B) and in PD (Figure 8C).

The lowest levels of binding were measured for [^18^F]fallypride in all three groups of subjects, suggesting a low dopamine D2/D3 receptor availability in the anterior cingulate. There was no significant difference between the CN and AD as well as PD subjects suggesting no change in dopamine receptors in AD and PD anterior cingulate.

## 4. Discussion

Our recent findings in the postmortem AD and PD anterior cingulate of subjects listed in Table 1 using [^18^F]FAZIN3 found significant increases in MAO-A binding [26,27]. Recent studies have also shown elevated levels of the isozyme MAO-B in AD [43]. Since both MAO-A and MAO-B are known to deaminate monoaminergic neurotransmitters, depleting essential neurotransmission and causing oxidative stress, and a plausible downstream effect on the serotonin receptors and dopamine receptors may be expected [44]. Because the cingulate cortex is one of the brain cortical regions affected by Aβ plaques, NFTs, and other proteinopathies [43,44], this study evaluated serotonin 5HT_1A_ and dopamine D2/D3 receptors in the anterior cingulate in postmortem AD and PD subjects.

Our results show a significant increase in the availability of serotonin 5HT_1A_ receptors in both AD and PD. This upregulation may be due to a plausible change in the neurotransmitter levels or other neuroinflammation processes. The increase in AD was 30%, while PD exhibited an increase of 11% of [^18^F]mefway binding. The increases of [^18^F]FAZIN3 binding to MAO-A were 50% in AD [26] and 59% in PD [27]. This increased MAO-A may play a role in the depletion of serotonin levels in the anterior cingulate brain slices in the present study. Previous studies of 5HT_1A_ receptors using various radiotracers have been mixed. It should be noted that MAO-A PET studies in depression showed higher levels of [^11^C]harmine binding in the anterior cingulate [45]. However, findings of 5HT_1A_ receptors have been mixed. Although a positive correlation between [^18^F]FAZIN3 binding and [^18^F]mefway binding was seen in both AD (Figure 8B) and PD (Figure 8C), a direct impact of MAO-A increases on increased 5HT_1A_ receptor binding will require further studies. In the limited studies of 5HT_1A_ receptors in AD and PD subjects, PET and postmortem studies have been inconclusive regarding changes in 5HT_1A_ receptors. Recent work on serotonin transporters (5-HTT) in MCI subjects shows a decrease in the binding of [^11^C]DASB [46], suggesting either an increase in synaptic serotonin levels or a down-regulation of 5-HTT in order to compensate for a decrease in synaptic serotonin.

Dopamine receptors have been extensively studied using PET imaging [47]. Much work has been performed on dopamine D2 receptors (D2R) in the striatum with the finding of an initial upregulation followed by a downregulation in about 4 years after the onset of motor symptoms. Using [^18^F]fallypride, brain regions outside the striatum were investigated in PET studies of PD subjects with motor and nonmotor symptoms [48]. A reduction in D2R was observed in the PD subjects in several brain regions, including the globus pallidus, caudate, amygdala, hippocampus, ventral midbrain, and thalamus. The anterior cingulate was not included in the analysis, plausibly because of the low levels of [^18^F]fallypride binding in this brain region [49,50]. Cognitive performance and cost–benefit decision making in healthy volunteers suggested possible modulation by dopamine D2/D3 receptors in the anterior cingulate cortex [51,52]. A decrease in dopamine D2/D3 receptors has been indicated in the anterior cingulate cortex in schizophrenia [53]. It has been suggested that a role of dopamine in AD may be associated with cognitive decline, and the use of dopamine agonists may help to improve cognitive functions [54]. Since executive functioning is compromised in both AD and PD, this preliminary study sought to evaluate any potential changes in dopamine D2/D3 receptors using [^18^F]fallypride.

This imaging study demonstrated that [^18^F]mefway binding to serotonin 5HT_1A_ receptors in AD and PD brains is elevated compared to CN brains in the anterior cingulate. On the other hand, [^18^F]fallypride binding to dopamine D2/D3 receptors in both AD and PD anterior cingulate compared to CN brains is not altered. This suggests that there is a differential effect between the two neurotransmitter-receptor systems, serotonin and dopamine, in both AD and PD. Thus, there may be a role for serotonergic drugs in AD [55,56] and among various treatments for PD [57].

Limitations of the study include the small number of subjects in advanced stages of AD and PD. A larger study with more subjects at different disease stages is needed to ascertain the receptor changes with the progression of disease and a possible role of neuroinflammation [58,59]. Our study here reports only one brain region; other brain regions need to be assessed. It must also be noted that depressive behaviors are often present in AD and PD, although our cohort of subjects in this study were not diagnosed with depression. Finally, increases in [^18^F]mefway binding in AD and PD subjects will have to be further confirmed by a more detailed Scatchard plot analysis, which will allow measurements of dissociation constant, K_D_, and receptor concentration, B_max_, of 5HT_1A_ receptors.

## 5. Conclusions

Our results showed significantly greater 5HT_1A_ receptor availability in the anterior cingulate of AD and PD subjects compared to CN subjects. This upregulation of 5HT_1A_ receptor availability may be a useful complementary biomarker for AD and PD. It also supports the need for in vivo PET imaging studies using [^18^F]mefway and [^18^F]FAZIN3 in order to ascertain the association between the two biomarkers. A larger study with more subjects at different disease stages and in other brain regions is needed in order to further assess the role of 5HT_1A_ receptors in neurodegeneration.

## Figures and Tables

**Table 1:** Patient Samples and Data *.

Subjects, N	CERAD Pathology	Gender	Age Range, Mean ± SD	PMI, hrs	Brain Region ^1^	Plaque Total	Tangle Total	LB	Braak Score
6	CN	5 Male 1 Female	73–92 (85.2 ± 7.03)	2–5.4	FC	0–5.5	0–6	0	II-III
6	AD	3 Male 3 Female	75–90 (81.3 ± 6.12)	1.8–5	FC	10–15	12–15	0	V-VI
6	CN	4 Male 2 Female	71–97 (79.9 ± 8.55)	2–5.4	AC	0–5.5	0–6	0	I-III
6	AD	5 Male 1 Female	70–91 (80.4 ± 5.98)	2.3–4.8	AC	14–15	10–15	0	V-VI
6	PD	4 Male 2 Female	53–95 (80.4 ± 13.1)	2.1–4.8	AC	0–10	0.5–6.5	0	I-III

* Frozen brain samples were obtained from Banner Sun Health Institute, Sun City Arizona described previously [26,32,40,41]; CN = cognitively normal and may include mild-cognitive-impairment (MCI) subjects; AD = Alzheimer’s disease; PMI: postmortem interval in hours; LB = Lewy bodies. ^1^ FC: frontal cortex and AC: anterior cingulate; brain slices (10 μm thickness) were obtained from frozen tissue and collected on Fisher slides.

## Data Availability

The data that supports the findings of this study are available from the corresponding author for discussions upon reasonable requests.
